# The Application of Fully Immersive Virtual Reality on Reminiscence Interventions for Older Adults: Scoping Review

**DOI:** 10.2196/45539

**Published:** 2023-10-06

**Authors:** Zhipeng Lu, Wenjin Wang, Wei Yan, Chung Lin Kew, Jinsil Hwaryoung Seo, Marcia Ory

**Affiliations:** 1 Department of Architecture, Texas A&M University College Station, TX United States; 2 School of Public Health, Texas A&M University College Station, TX United States; 3 School of Performance, Visualization & Fine Arts, Texas A&M University College Station, TX United States

**Keywords:** older adults, fully immersive virtual reality, reminiscence, Alzheimer, cognitive function, mental health, psychological well-being, memory care, dementia, scoping review

## Abstract

**Background:**

The increasing number of older adults with mental, behavioral, and memory challenges presents significant public health concerns. Reminiscence is one type of nonpharmacological intervention that can effectively evoke memories, stimulate mental activities, and improve psychological well-being in older adults through a series of discussions on previous experiences. Fully immersive virtual reality (FIVR) may be a useful tool for reminiscence interventions because it uses realistic virtual environments connected to a person’s significant past stories.

**Objective:**

This review aims to examine empirical evidence regarding the application of FIVR in reminiscence interventions, its usability and acceptability, and its effectiveness in assisting the intervention to achieve optimal outcomes.

**Methods:**

We followed the PRISMA (Preferred Reporting Items for Systematic Reviews and Meta-Analyses) approach for scoping reviews. The PubMed, PsycINFO, Embase, CINAHL, Web of Science, ACM, and IEEE Xplore electronic databases were used for the search. We included peer-reviewed studies that used FIVR as an assistive tool for reminiscence interventions; were published between January 1, 2000, and August 1, 2022; reported empirical research; involved older adults as participants; and addressed health- and behavior-related outcomes or the feasibility and usability of FIVR. We used Endnote X9 to organize the search results and Microsoft Excel for data extraction and synthesis.

**Results:**

Of the 806 articles collected from the databases and other resources, 11 were identified. Most of the studies involved participants aged between 70 and 90 years. Only 1 study did not involve those with cognitive impairments, whereas 3 specifically targeted people living with dementia. The results indicated that FIVR reminiscence interventions enhanced engagement and reduced fatigue. Although some studies have observed positive effects on anxiety, apathy, depression, cognitive functions, and caregiver burden reduction, these findings were inconsistent across other research. In addition, FIVR showed overall usability and acceptability with manageable side effects among older adults across various health conditions during reminiscence sessions. However, 1 study reported adverse feelings among participants, triggered by unpleasant memories evoked by the virtual reality content.

**Conclusions:**

The role of FIVR in reminiscence interventions remains nascent, with limited studies evaluating its impacts on older adults. Many of the reviewed studies had notable limitations: small sample sizes, absence of rigorous research design, limited assessment of long-term effects, lack of measures for health and behavior outcomes, and quality of life. Beyond these limitations, this review identified a list of future research directions in 6 categories. On the basis of the review findings, we provide practical recommendations to enhance FIVR reminiscence interventions, covering topics such as virtual reality content, device choice, intervention types, and the role and responsibility of facilitators.

## Introduction

### Background

In recent years, there has been a surge in groundbreaking technologies and their applications in transforming care, therapies, treatments, and health prevention for older adults. Fully immersive virtual reality (FIVR) is one among these rapidly evolving technologies applied in diverse interventions, including poststroke rehabilitation [1], cognitive training for people with neurodegenerative diseases [2], mobility and balance training [3], and the reduction of depression [4]. This paper examines the application of FIVR in reminiscence interventions through a scoping literature review, with a particular focus on people living with dementia or mild cognitive impairments.

### Reminiscence Interventions

Reminiscence intervention is a type of nonpharmacological therapy [5] that was developed in the 1960s and has gained popularity since the 1980s [6]. Reminiscence is an act of recalling or retelling past experiences or facts [7]. As an early advocate of this technique, Butler [8] argued that reminiscence was an older person’s natural adaptive response to late-life developmental crises, with thoughts related to their approaching death. Used in life review therapy, reminiscence helps an older person reorganize past experiences, settle unresolved issues, bring new meaning to the present life, and better prepare for death [8]. Molinari [9] expanded on the notion proposed by Butler [8] and purported that reminiscence activities could take many forms, including storytelling, autobiography, music, reunions, and scrapbooks. He suggested that reminiscence could be conducted in community or clinical settings, with a person or group of people, by laypersons, or by trained professionals; with structured or unstructured methods; or in a formal or casual format [9]. However, many states in the United States, such as Texas, require counseling therapy to be delivered by licensed professionals [10]; interventions done by family members, friends, and noncertified personnel may be referred to as informal exercises or activities.

Pinquart and Forstmeier [11] and Woods et al [12] identified three main types of reminiscence interventions: (1) simple reminiscence, which involved the recall and sharing of positive memories and stories to increase positive feelings; (2) life review, which was conducted in a structured way with the whole-life story, seeking to integrate both negative and positive memories; and (3) life review therapy, which typically aimed at the reevaluation of negative memories, promoting a more positive view of life.

According to Molinari [9], reminiscence interventions help reshape a person’s views of life experiences and stories toward positive and coherent outcomes, focusing on strength, success, and lessons learned. Therefore, reminiscence interventions have been used in diverse settings for various purposes: in dementia and Alzheimer care to lessen behavioral and psychological symptoms and enhance cognitive function, social interaction, self-esteem, and quality of life [12,13]; in end-of-life care, such as hospices and palliative care, to enhance life satisfaction and reduce feelings of regret [14]; in patients with cancer to lower anxiety and depression scales and increase hope, dignity, and quality of life [15]; and in rehabilitation to motivate patients to actively participate in the rehabilitation process, fostering a sense of identity and continuity in their life story [16]. Moreover, a literature review conducted by Shin et al [17] revealed that a reminiscence intervention was effective in improving the quality of life and life satisfaction among community-dwelling older adults with no dementia symptoms.

### Cues for Reminiscence Interventions

Similar to other psychotherapeutic interventions, a reminiscence session is primarily carried out through conversations, with a provider guiding the person to recall past events, discuss their meanings and impacts, resolve past conflicts, and pave the way for more positive views of the present life. Various media or artifacts that provide visual or audio cues have been applied to stimulate memory and prompt conversations, especially for those with memory problems.

In a literature review, Lazar et al [18] reported that common media for reminiscence interventions included text, videos, photographs, images, music, audio, and personal objects and artifacts such as toys, which were closely related to the person’s past experience. The authors also revealed that the technologies to present these media ranged from slide projectors and tape recorders from the 1990s to touchscreen tablets in the early 2010s [19-21]. Moreover, digital technologies have since created more innovative alternatives to aid reminiscence [22-25]. For example, personalized digital memory books and multisensory mobile multimedia environments delivered more content or multiple stimuli in convenient ways [26].

**Figure 1:**
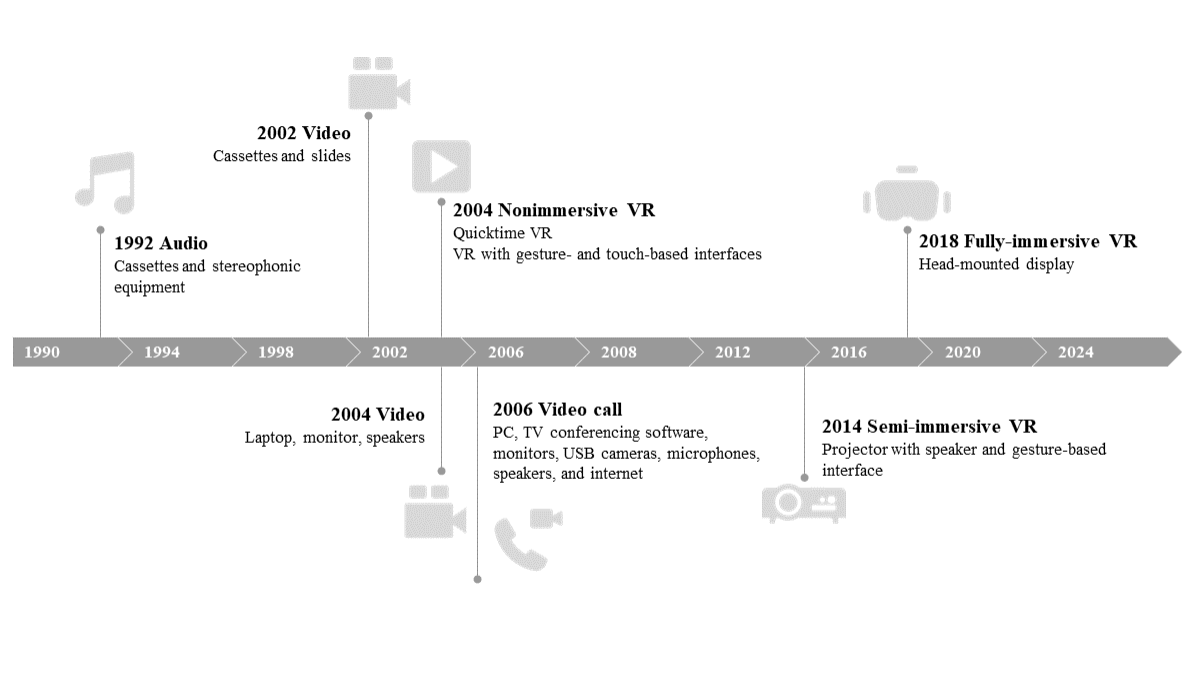
Timeline of the technologies applied to reminiscence interventions documented in peer-reviewed publications. VR: virtual reality.

Figure 1 [19-25,27] shows the timeline of different technologies used in reminiscence interventions since 1992. It is worth noting that therapists may have used technologies to aid reminiscence activities in earlier years, but this was not documented in peer-reviewed publications.

### Virtual Reality as an Aid to Reminiscence

Virtual reality (VR) uses computer simulation to provide realistic visual and tactile experiences in a 3D virtual world. VR appears in many forms: (1) nonimmersive, in which a computer or television screen is used to display the content; (2) semi-immersive, in which multiple screens or a Cave Automatic Virtual Environment display present the virtual environment surrounding the participant [28]; and (3) fully immersive, in which the participant wears the VR head-mounted display or haptic devices to be fully immersed in the virtual world. According to Logan [29], VR possesses three main characteristics: (1) interactivity, whereby the user can interact with the virtual world through commands; (2) immersion, whereby the user should have similar authentic feelings, through different sensors, such as those in real environments; and (3) imagination, whereby the user should be able to imagine beyond the virtual world, finding new perspectives or new ways to solve problems.

VR has been applied in a variety of interventions for older adults. D’Cunha et al [30] conducted a literature review on the application of VR and augmented reality in nonpharmacological interventions, examining their effectiveness in promoting psychological health and users’ experience. Three types of interventions were tested in the reviewed studies: (1) cognitive or memory training; (2) reminiscence; and (3) therapeutic activities, including physical exercises. The results showed that VR and augmented reality were preferred and well accepted by people living with dementia or mild cognitive impairment and that these technologies helped improve mood, apathy, and cognitive functions. However, some side effects raised concerns about negative feelings after the reminiscence sessions that triggered participants’ unpleasant memories. Although immersive VR was used in some studies selected by the authors, most of them were semi-immersive but did not use a head-mounted display that would provide a fully immersive experience.

Although the first head-mounted VR display with 3D graphics and head tracking was invented in the 1960s [31], it was not until the last decade, marked by the introduction of the first consumer-grade VR headset (Oculus Rift) in 2011, that FIVR became more affordable, more technically accessible, and easier to use [32]. FIVR has been popular in a variety of fields such as gaming, education, professional training, medical practice, therapies, and research [33-37]. With its capability to promote interactivity, immersion, and imagination, FIVR may be an excellent aid for reminiscence interventions by stimulating or reactivating the past memories of older adults, especially those living with mild cognitive impairment or dementia.

### Reminiscence Interventions for People Living With Dementia or Mild Cognitive Impairments

Population aging has brought about a rapid increase in the number of older adults with cognitive and behavioral problems. Rajan et al [38] estimated that 12.23 million Americans aged ≥65 years experienced mild cognitive impairments in 2020 and that this number would rise to 21.55 million in 2060. The Alzheimer’s Association reported that 10% to 15% of people with mild cognitive impairment transitioned to dementia each year [39]. Furthermore, approximately 1 in 9 older adults (10.7%) in the United States had Alzheimer disease, accounting for 60% to 80% of people living with dementia. Cognitive impairment affecting memory, language, orientation, attention, and judgment leads to behavioral and psychological disturbances, dysfunction in activities of daily living, and instrumental activities of daily living [40-42]. Such functional disabilities and behavioral problems have added to caregivers’ and family members’ burdens and strained health care resources [43].

According to the Alzheimer’s Association, pharmacological and nonpharmacological options may be available to treat cognitive impairments, depending on their causes [39]. However, pharmacological treatment options are limited, with only 6 drugs—specifically for Alzheimer disease—currently approved by the US Food and Drug Administration [39]. In addition, there is evidence that pharmacological treatments for behavioral and psychological symptoms of people living with dementia often come with adverse side effects, including an increased risk of falls and fractures, stroke, and even mortality and limited efficacy in symptom management [44]. Nonpharmacological therapies are safer options with manageable side effects for people with cognitive impairments than pharmacological treatments. These therapies can help reduce anxiety, depression, apathy, irritation, and other negative emotions and possibly decrease the need for pain and psychoactive medications by not inducing biological changes [45]. After reviewing the literature, Cammisuli et al [5] categorized nonpharmacological interventions into four main categories: (1) holistic techniques, including reality orientation that helps people living with dementia be aware of who and where they are and what time it is; cognitive stimulation therapy that uses a series of objects, themes, or activities, such as physical games, sounds, food, current affairs, scenes, word association, using money, and number games to stimulate cognitive functioning; and reminiscence; (2) brief psychotherapies, a type of psychodynamic therapy that emphasizes the identity of people living with dementia through a conversation about past experience, with the aim of reducing emotional distress and other behavioral problems; (3) cognitive methods, such as the space retrieval technique, which trains people living with dementia to regain the ability to associate names with human faces or with objects; and (4) alternative strategies, such as music therapy and bright light therapy [5].

As previously noted, Cammisuli et al [5] categorized reminiscence interventions as a holistic technique of nonpharmacological therapy. Evidence has shown that appropriate reminiscence interventions for older adults with cognitive impairment can effectively evoke memories, alleviate anxiety and depression, reduce stress, manage cognitive decline, increase self-esteem, and improve quality of life [11,46,47]. Importantly, these interventions may potentially lower caregivers’ burden and stress due to the improvement in their care recipients’ psychological, mental, and behavioral health [48], which is significant as the world is experiencing a serious shortage in the nursing workforce.

### Research Aims

We found little collective knowledge on how FIVR works in reminiscence interventions. Therefore, this scoping review aimed to assess and synthesize existing evidence regarding the influence and effectiveness of FIVR as an aid in reminiscence interventions. We expected the findings to elucidate potential directions for future research, offer recommendations for practice, and address the critical needs of caring for frail older adults, particularly those living with dementia and mild cognitive impairments.

## Methods

### Overview

We adopted PRISMA-ScR (Preferred Reporting Items for Systematic Reviews and Meta-Analysis extension for Scoping Reviews) to map the evidence for FIVR-based reminiscence interventions. PRISMA (Preferred Reporting Items for Systematic Reviews and Meta-Analyses) is a minimum set of evidence-based items for reporting in systematic reviews and meta-analyses [49]. The PRISMA-ScR—with 20 essential reporting items and 2 optional items—was created to synthesize evidence and assess the scope of the literature on a topic [50]. The detailed descriptions of the 22 items are reported in Multimedia Appendix 1. Compared with systematic reviews, scoping reviews address a broader research question [51]. They allow researchers to identify the scope of the literature in a certain area, provide a clear indication of the available literature and an overview of its focus, and examine emerging evidence that informs field practice [51]. We adopted the scoping review approach because it perfectly aligns with our study’s aims.

### Search, Screening, and Selection

We applied the following inclusion and exclusion criteria for article selection: (1) the use of FIVR as an aid for reminiscence interventions; (2) the inclusion of adults aged ≥65 years; (3) reporting on empirical studies; (4) reporting on outcomes related to health and well-being and the feasibility or usability of FIVR; (5) publication in English; and (6) publication in peer-reviewed journals or peer-reviewed conference proceedings between January 1, 2000, and August 1, 2022. Electronic databases—PubMed, PsycINFO, Embase, CINAHL, Web of Science, ACM, and IEEE Xplore—were used for the literature search. The search terms included all possible synonyms and combinations of “Reminiscence Interventions and/or Therapies” (reminiscence, life story, reminisc* therapy*, and memory simulation) and “Virtual Reality” (VR, imagined 3D environment, and simulat* environment).

First, a researcher used a consistent set of keywords to search each database between March and August 2022. The search results were imported and organized using Endnote X9 (Clarivate), a computer reference management program. Second, 2 researchers scanned the titles and subsequently the abstracts of the retrieved articles. They then independently reviewed the full texts, collating all eligible articles. References from these articles were also scrutinized to capture any potentially overlooked information in the initial database search. Subsequently, the researchers deliberated each article in detail, deciding on its inclusion based on predetermined criteria and the study objectives. In case of any disagreements between the 2 researchers, a third researcher was ready to intervene with a final decision. However, this contingency was not triggered.

### Data Extraction and Analysis

We continued to use Endnote X9 to manage the selected articles and Microsoft Excel spreadsheets for data extraction. The categories of the data included title, author, year of publication, publication type such as journal or conference paper, research design, research questions, intervention information including reminiscence types, VR contents, VR types, and devices, and participant information, measurements, results, study limitations, and future research questions. One researcher charted the data into a spreadsheet, whereas another oversaw the process, critically assessed the extraction, and made necessary adjustments based on joint discussions when a disagreement arose. The charted data were further summarized and analyzed by both researchers.

## Results

### Search Results

**Figure 2:**
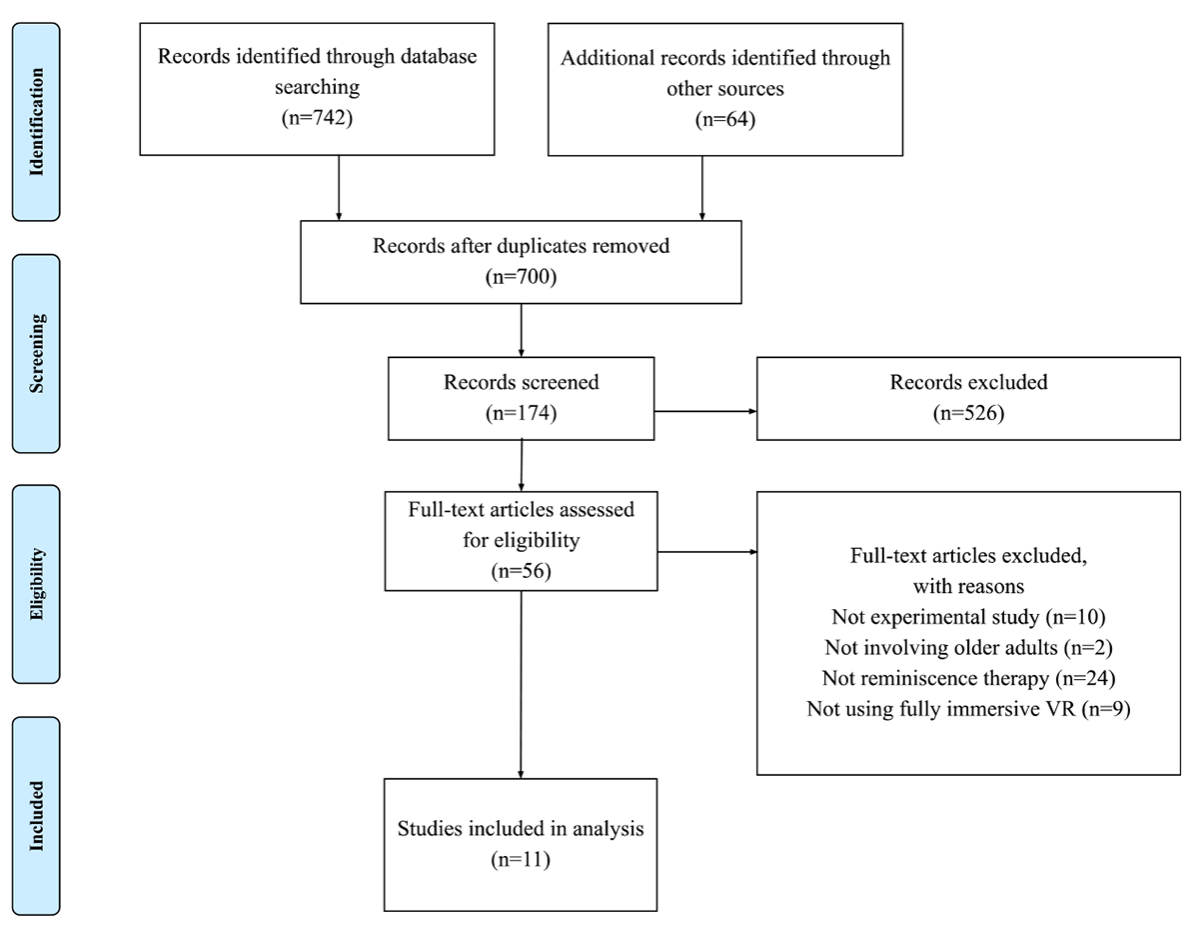
PRISMA (Preferred Reporting Items for Systematic Reviews and Meta-Analyses) flow diagram of the literature search and screening process. VR: virtual reality.

The screening and selection process for eligible articles is shown in Figure 2. Additional details can be found in Multimedia Appendix 2. The first search resulted in 742 articles from the databases and 64 others from further sources such as references of selected articles. After removing the duplicates, 700 articles were retained. After the authors screened each article’s title and abstract, 174 articles remained in the pool. We quickly scanned the full text and retained 56 articles for additional evaluation. Through the detailed full-text reviews, 45 studies were excluded according to the inclusion criteria, with specific reasons documented in Figure 2. Finally, 11 studies were identified for this scoping review [25,40,52-60].

### Characteristics of the Selected Studies

Table 1 summarizes the general information of the selected studies, including basic article information such as authors, year of publication, and research design.

Nine articles were published in peer-reviewed journals and 2 in peer-reviewed conference proceedings. There were 4 studies that were conducted in Australia [53,56-58], 4 in Asia (China, Taiwan, and Japan) [54,55,59,60], 2 in Europe (Germany and Portugal) [25,40], and 1 in the United States [52]. The years of publication were between 2018 and 2022.

**Table 1:** General information summaries of selected studies.

Study, year	Publication type (study location)	Research design
Afifi et al [52], 2021	Journal paper (United States)	Mixed methods
Baker et al [53], 2021	Conference paper (Australia)	Qualitative research
Coelho et al [40], 2020	Journal paper (Portugal)	Mixed methods
Huang and Yang [54], 2022	Journal paper (Taiwan)	Quantitative research
Klein et al [25], 2018	Journal paper (Germany)	Qualitative research
Niki et al [55], 2020	Journal paper (Japan)	Quantitative research with randomized crossover research design
Saredakis et al [56], 2020	Journal paper (Australia)	Mixed methods
Saredakis et al [57], 2021	Journal paper (Australia)	Quantitative research with multisite nonrandomized controlled trial
Webber et al [58], 2021	Journal paper (Australia)	Qualitative research
Xu and Wang [59], 2020	Conference paper (China)	Randomized controlled trial
Yahara et al [60], 2021	Journal paper (Japan)	Case study

### Participant Characteristics

Table 2 lists the participant characteristics of the included studies. All the studies involved older participants, with an average age between 80 and 90 years in 6 studies and 70 and 80 years in 3 studies. Two articles did not specify the participants’ ages. All but one study included participants with cognitive impairments. Three studies specifically stated that they recruited people living with dementia [40,53,59]. Three studies reported other health problems in their participants, including depression, anxiety, heart diseases, mental health issues, mobility challenges, hearing difficulty, Parkinson disease, and stroke [54,56,57].

**Table 2:** General participant information.

Study, year	Sample size, gender, and age	Cognitive or dementia conditions	Other health conditions
Afifi et al [52], 2021	21 residents (female: n=18, male: n=3; mean age 83.1, SD 3.72 y) with their family members (female: n=9, male: n=12; mean age 59.86, SD 14.12 y)	Mild cognitive impairment (n=9) Mild dementia (n=4) Moderate dementia (n=8)	Not specified
Baker et al [53], 2021	16 older adults (female: n=5, male: n=11)	Not specified	Hearing difficulty (n=4) Need mobility aid (n=2) Mental health problem (n=1) Parkinson disease with communication or movement problem (n=1)
Coelho et al [40], 2020	9 individuals (female: n=6, male: n=3; mean age 85.6, SD 7.4 y)	Dementia (n=9) Moderate cognitive decline (n=3) Moderately severe cognitive decline (n=3) Severe cognitive decline (n=3)	Not specified
Huang and Yang [54], 2022	20 participants (female: n=11, male: n=9; mean age 79.0, SD 7.8 y) Among the 20 participants, 7 were assessed 3-6 mo after the intervention	Very mild dementia (n=2) Mild dementia (n=15) Moderate dementia (n=3)	Not specified
Klein et al [25], 2018	6 participants (female: n=3, male: n=3; mean age 74.67, SD 1.31 y)	Dementia (n=4) Cognitive impairment without an unequivocal diagnosis of dementia (n=2)	Not specified
Niki et al [55], 2020	10 individuals (female: n=6, male: n=4; mean age 87.1, SD 4.2 y)	Normal cognition (n=9) Suspected mild cognitive impairment (n=1)	Not specified
Saredakis et al [56], 2020	17 participants (female: n=10, male: n=7; mean age 87.3, SD 6.3 y)	No or minimal cognitive impairment (n=10) Mild cognitive impairments (n=3) Moderate cognitive impairments (n=4)	Depressive symptoms (n=6)
Saredakis et al [57], 2021	Experiment group: 15 participants (female: n=10, male: n=5; mean age 81.7, SD 6.6 y) Active control: 14 participants (female: n=9, male: n=5; mean age 85.9, SD 8.1 y) Passive control: 14 participants (female: n=9, male: n=5; mean age 87, SD 8.7 y) Total: 43 participants (female: n=28, male: n=15; mean age 84.8, SD 8 y)	Memory-related dementia or Parkinson disease (n=5) Memory-related dementia or Parkinson disease (n=3) Memory-related, dementia or Parkinson disease (n=3) Memory-related dementia or Parkinson disease (n=11)	Depression (n=5) Anxiety (n=5) Heart disease (n=8) Stroke (n=2)
Webber et al [58], 2021	6 participants + 1 participant in pilot study	With signs of cognitive impairment (n=4)	Not specified
Xu and Wang [59], 2020	Virtual reality–based group: 10 participants (female: n=5, male: n=5; mean age 76.7, SD 5.5 y) Photo-based group: 10 participants (female: n=8, male: n=3; mean age 79.4, SD 2.0 y) Blank group (verbally guided): 10 participants (mean age 78.5, SD 3.4 y)	Mild to moderate dementia	Not specified
Yahara et al [60], 2021	2 participants (female: n=1, male: n=1; age 80 and 92 y)	Mild cognitive impairment (n=2)	Not specified

### Research Designs

There was 1 randomized crossover trial [55] and 1 randomized controlled trial [59]. Two quasi-experiments with pre-post comparisons were included [56,57], one of which had a control group. Another quasi-experiment was conducted with pre-post comparisons, wherein the intervention was offered twice a week over a 3-month period. Assessments were performed immediately and 3 to 6 months after the intervention [53]. The remaining studies included 3 qualitative studies [30,43,47], 2 mixed methods studies [40,52], and 1 case study [60]. The sample sizes ranged from 2 to 43.

### FIVR Instruments and Stimuli

Tables 3 and 4 summarize the VR-related information of the selected studies, including VR types and visual contents, outcome measures, measurement methods, and results.

**Table 3:** Virtual reality (VR) types and visual contents used in the selected studies.

Study, year	VR type	Visual content
Afifi et al [52], 2021	Photo based Video based Computer graphic based	Virtual adventures Virtual life story Virtual photos and videos
Baker et al [53], 2021	Computer graphic based	School hall and classroom
Coelho et al [40], 2020	Video based	Specific streets, squares, gardens, churches, and historical landmarks that were meaningful to participants
Huang and Yang [54], 2022	Computer graphic based (with customized narration and music that was significant to the person)	Historical type of residence commonly found throughout Taiwan from 1960-1980 Interactive features: the participant could use controller to hold rice to fee chickens
Klein et al [25], 2018	Photo based Video based	Time travel (Berlin, 1949-1970; Paris, 20th century), movie stars (1950-1960), Germany television shows (later 20th century), or handicraft images
Niki et al [55], 2021	Photo based Computer graphic based	A theme park with 6 familiar situations to those aged ≥75 y
Saredakis et al [56], 2020	Photo based	Personal tailored content
Saredakis et al [57], 2021	Photo based	Personal tailored content (not specified)
Webber et al [58], 2021	Photo based	Personal nominated content
Xu and Wang [59], 2020	Computer graphic based	A Chinese rural cottage in the 1970s with an area of 30 m2
Yahara et al [60], 2021	Photo based	Personal memorable places (not specified)

**Table 4:** Measures, measurement instruments, and results.

Study, year	Measure	Measurement instruments	Results
Afifi et al [52], 2021	User satisfaction and perceptions Conversational and behavioral engagement Kinesics engagement	Self-report questionnaire Coding analysis of video recording Coding analysis of video recording	VR^a^ deemed to be safe, extremely enjoyable, and easy to use by residents and family members Residents being more conversationally and behaviorally engaged with their family members in VR sessions than in the baseline telephone calls Residents with dementia reporting greater immersion in the VR than residents with MCI^b^ Residents with MCI being more kinetically engaged while using the VR than residents with dementia.
Baker et al [53], 2021	Older adults’ ability to participate	Questionnaire, interviews, screen-captured video, observation notes, photographs, and short video recordings	The virtual environment playing an effective role in surfacing memories and scaffolding reminiscence
Coelho et al [40], 2020	Engagement and behavior Manifestation of psychological and behavioral symptoms Specific symptoms associated with simulation experiences Psychological and behavioral symptomatology and the quality of life Caregivers’ opinion	Coding analysis of observation Coding analysis of observation Self-report questionnaire Structured interview with a knowledgeable informant Semistructured interview	Participants being very interested in exploring the immersive virtual environment and addressing positive or happy memories with frequently spontaneous communications and without simulator sickness in most cases No significant differences found in psychological and behavioral symptomatology and the quality of life Potentially beneficial experience for most participants reported by caregivers
Huang and Yang [54], 2022	Overall cognitive function Cognitive impairment Dementia status Depression Caregiver burden	CASI^c^ MMSE^d^ CDR^e^ and its CDR-SB^f^ CESD^g^ ZBI^h^	No significant differences in cognition (MMSE and CASI), global status of dementia (CDR-SB), and caregiver burden (ZBI) scores before and immediately after the intervention No significant differences in MMSE, global status of dementia (CDR-SB), and caregiver burden (ZBI) scores immediately after the intervention and 3-6 mo after the intervention Significantly decreased cognitive abilities (CASI) 3-6 mo after the intervention compared with those immediately after the intervention Significantly improved depression (CESD) symptom immediately after the intervention Significantly improved depression (CESD) symptom 3-6 mo after the intervention, compared with those immediately after the intervention
Klein et al [25], 2018	“Destructive” and “supportive” behaviors Usability Caregivers’ recommendations and assessment of feasibility	Rating with a qualitative behavioral protocol Rating with ISO 9241-110 criteria Semistructured interview	The prototype fostering conversations between participants and caregivers, positive interactions, and more “supportive” behaviors (eg, recognition, celebration, relaxation, validation, and facilitation) than “destructive” behaviors (eg, outpacing, ignoring, and imposition). The prototype deemed to be suitable for daily care but being needed to be more fluently and with a bigger angle by caregivers
Niki et al [55], 2020	Anxiety Satisfaction and side effects	Self-report questionnaire Self-report questionnaire	VR reminiscence reducing anxiety without causing serious side effects. VR with live-action images being preferred by participants than computer graphics images
Saredakis et al [56], 2020	Apathy; side effect; realistic experience Verbal fluency Expectations or enjoyment Debriefing	Self-report questionnaire Task Self-report questionnaire	Enjoyable and acceptable experience with some negative symptoms or side effects reported by all study particiapnts Improved semantic scores but not phonemic fluency scores found in participants Greatest cognitive improvements after a VR reminiscence experience found in participants with higher levels of apathy
Saredakis et al [57], 2021	Apathy Cognition Depression; quality of life; loneliness; side effect Staff’s opinions Attendance and responses	Questionnaire Examination Self-report questionnaire Staff questionnaire Coding analysis of recordings	Most participants in the VR group preferring to watch content in VR than on a flat screen. Participants enjoying the process of reminiscence Participants reporting their willingness to do reminiscence again. No significant results observed for cognition, depression, quality of life, and loneliness after therapy No reported significant side effects or discomfort
Webber et al [58], 2021	Perceived value; varied forms of reminiscence; challenges Motivation and expectation	Observation, semistructured interviews, and questionnaire Interviews with family members	Various past memories being elicited for all participants with mixed feelings expressed by several participants. Virtual visits prompting various forms of reminiscence without noticeably differences compared with tablet computers. Diverse expectations about the potential value of virtual visits revealed by families
Xu and Wang [59], 2020	Acceptability (motivation, presence, and VR sickness) Autobiographical memory	Self-report questionnaire Rating with video recording	The VR group and the photo group with higher scores in recalling autobiographical memory than the blank group, with no significant difference between the VR group and the photo group Higher levels of interest, motivation and perceived security, and lower levels of anxiety and fatigue in participants in both the VR group and the photo group than those in the blank group Significantly higher level of pleasure reported in the VR group than those in the photo group. High levels of presence with negligible sickness symptoms in the VR group
Yahara et al [60], 2021	Anxiety; side effect and satisfaction; motivation The burden of care Attitude toward participation in the study	Self-report questionnaire Questionnaire by family members Coding analysis of observations and videos	Reduced anxiety and the burden of care without serious side effects by IVR^i^ reminiscence Similar effectiveness found between remote IVR reminiscence and the face-to-face session

^a^VR: virtual reality.

^b^MCI: mild cognitive impairment.

^c^CASI: Cognitive Abilities Screening Instrument.

^d^MMSE: Mini-Mental State Examination.

^e^CDR: Clinical Dementia Rating.

^f^CDR-SB: Clinical Dementia Rating–Sum of Boxes.

^g^CESD: Center for Epidemiological Studies Depression.

^h^ZBI: Zarit Caregiver Burden Interview.

^i^IVR: immersive virtual reality.

The VR goggles developed by Oculus were used in most studies (n=8): Oculus Go in 5 studies [52,55,56,58,60], Oculus Rift in 2 studies [40,54], and Oculus Quest in 1 study [57]. Coelho et al [40] used Samsung Gear VR as a head-mounted display in addition to the Oculus Rift. Xu and Wang [59] adopted the HTC VIVE Focus headset. Huang and Yang [53] used the HTC VIVE Pro version. Klein et al [30] developed a VR goggle—the “Binoculars” prototype—for their own project.

We divided FIVR stimuli into three categories: (1) photo based, which used 360° photos of real environments; (2) video based, which used 360° videos of real environments; and (3) computer graphic based, which used 360° images or animations of virtual environments generated by computer 3D modeling programs. Seven studies used photo-based VR stimuli [25,52,55-58,60]. Five studies used computer graphics–based VR, one of which incorporated recorded customized narrative and simple interactive functions via VR handles [52-55,59]. Two studies applied video-based VR [3,30], whereas 3 adopted ≥2 types of VR stimuli [25,52,55].

### Types of Reminiscence Interventions

Only the study conducted by Saredakis et al [57] specified the type of reminiscence intervention used: a semistructured simple reminiscence approach emphasizing positive memories. The 2 other possible approaches, “life review” and “life story review,” were not found in the rest of the articles.

Reminiscence interventions in the selected studies can be categorized based on the way they were conducted (group or individual) and the people involved (certified therapists, caregivers, or researchers). Eight studies were conducted using an individual format. Only the study by Baker et al [54] investigated how VR applications facilitated group reminiscence, in which participants communicated with each other and with a facilitator who was determined before the conversation started. During the interventions, 5 studies used researchers as facilitators [40,53,56-58]. One study used a professional team comprising pharmacists, an occupational therapist, a clinical psychologist, a certified psychologist, and a pharmacy student [60]. Interventions in 2 studies were conducted by researchers accompanied by caregivers or nursing staff [55,59]. All but 2 were conducted only through face-to-face meetings [52,60]. Participants in the study by Afifi et al [52] experienced the FIVR scenarios simultaneously with their family members who lived at a distance, interacting with each other remotely.

### Health-Related and Behavior-Related Outcomes

All studies reported health- and behavior-related outcomes after reminiscence interventions. For the participants, we identified seven types of health- and behavior-related outcomes reported in the studies: (1) anxiety, (2) apathy, (3) cognitive functions or dementia status, (4) engagement, (5) depression, (6) loneliness, and (7) fatigue.

#### Anxiety

Three studies examined the effect of FIVR reminiscence on anxiety [40,55,60]. Instruments for measuring anxiety included the State-Trait Anxiety Inventory Self-Report Questionnaire [55,60], the Cornell Scale for Depression in Dementia, and the Neuropsychiatric Inventory [40]. Niki et al [55] found that FIVR reminiscence interventions reduced anxiety in older adults after the first session, maintaining effectiveness after the second [55]. They discovered that 360° photos are more effective than computer-generated graphics. Yahara et al [60] compared face-to-face and remote FIVR reminiscence sessions in 2 participants with mild cognitive impairments. Both participants experienced significantly decreased anxiety after the face-to-face sessions. However, after shifting to the remote session, 1 participant displayed a slight increase in anxiety, possibly due to the difficulty in remote communication, whereas the other participant’s anxiety level continued to decrease. In the study by Coelho et al [40], some participants exhibited mild or intermittent anxiety and agitated behaviors during VR reminiscence sessions, which might have been caused by the disruption of participants’ daily routines and unfamiliarity with the experiment environment [40].

#### Apathy

Apathy is defined as a “lack of feeling or emotion” or a “lack of interest or concern” [61]. Four studies assessed participants’ changes in apathy after FIVR reminiscence interventions [40,56,57,60]. Saredakis et al [56,57] evaluated the effect of FIVR reminiscence therapy on apathy through verbal fluency and the Apathy Evaluation Scale. Verbal fluency, as noted by the authors, demonstrates the capability for executive control and initiation. The decline in these executive functions was associated with decreased apathy. They found that participants had improved semantic scores—the ability to name as many words as possible, starting with specific letters but not phonemic fluency scores and the ability to list more words in the category of either animals or fruit or vegetables—after the reminiscence session [56]. In another study conducted by the same team, there was an improvement in participants’ apathy scale scores after reminiscence interventions [57]. Using the Cornell Scale for Depression in Dementia and the Neuropsychiatric Inventory, Coelho et al [40] found no significant improvement of apathy. Yahara et al [60] assessed participants’ motivations and found that the effects of FIVR reminiscence on participants’ apathy levels were mixed.

#### Cognitive Functions and Dementia Status

Three studies assessed the effects of the FIVR reminiscence intervention on participants’ cognitive functions or dementia status, using measurement tools including the Cognitive Abilities Screening Instrument, the Mini-Mental State Examination, the Clinical Dementia Rating, the Addenbrooke Cognitive Examination III, and the Psychogeriatric Assessment Scales [53,56,57]. Two studies reported nonsignificant findings [56,57]. Huang and Yang [53] noted no significant cognitive ability differences before and immediately after the intervention, but the scores significantly declined 3 to 6 months later, implicating that the intervention might help sustain participants’ cognitive functions [53]. In addition, Saredakis et al [56] observed that participants with higher apathy levels showed greater cognitive improvement after the intervention.

#### Engagement

Engagement was evaluated in 3 studies through observations [25,40,52]. Afifi et al [52] stated that participants were more engaged in conversations and behaviors with their family after FIVR reminiscence sessions. Those with mild cognitive impairments exhibited more kinetic engagement than people living with dementia [52]. Coelho et al [40] and Klein et al [25] found that FIVR reminiscence interventions encouraged conversation and positive participant-caregiver interactions.

#### Depression

Depression was measured using the Center for Epidemiological Studies Depression and the Geriatric Depression Scale (Short Form) in 2 studies [53,57]. Huang and Yang [53] noted that participants experienced significant depression improvements immediately and 3 to 6 months after the intervention. In contrast, Saredakis et al [57] reported no significant influence on depression.

#### Loneliness

One study assessed intervention effects on loneliness using the Three-Item Loneliness Scale, finding no significant outcomes [57].

#### Fatigue

One study reported less fatigue in the reminiscence sessions supported by FIVR and photos, compared with the control group with no visual aids [59].

### Quality of Life

Two studies concluded that FIVR reminiscence interventions did not significantly affect participants’ perceived quality of life, as assessed by the European Health Interview Survey–Quality of Life (EUROHIS-QOL) 8-item Index and the Quality of Life in Alzheimer Disease 13-item Scale [40,57].

### Caregivers’ Burdens

Caregivers’ burdens were examined in 2 studies. Yahara et al [60] found that the FIVR reminiscence intervention reduced caregivers’ burdens, but Huang and Yang [53] reported no significant differences in the caregiver burden scores before and after the intervention.

### Usability and Acceptability

The usability and acceptability of FIVR in reminiscence sessions were evaluated using various methods such as a self-report questionnaire [55], behavior observation and analysis [25,40,52], and interviews [58]. Most participants indicated that FIVR reminiscence effectively evoked their past memories and was both enjoyable and acceptable. However, 1 study reported that FIVR triggered unpleasant memories in several participants [58].

Of the 4 studies addressing adverse effects, Saredakis et al [56] reported “some” negative symptoms or side effects. In contrast, the other 3 studies found “no simulator sickness” [40], “no significant side effects/discomfort” [57], and “negligible sickness symptoms” [59].

Four studies included caregivers and family members as participants [25,40,58,60]. Both Coelho et al [40] and Webber et al [58] observed that caregivers and family members viewed the FIVR reminiscence experience as potentially beneficial and valuable. However, 1 study [25] noted that caregivers found the reminiscence intervention impractical in an ambulant daycare setting because of the lack of staff for individual sessions.

### Comparison Between FIVR and Other Technologies

Three studies compared different effects between the applications of FIVR and other technologies in reminiscence sessions [57-59]. Two studies found that the use of FIVR and a flat screen for reminiscence did not result in noticeable differences among participants [57,58], although participants in the study by Saredakis et al [57] preferred the FIVR experience over a flat screen. Xu and Wang [59] reported that both FIVR and printed photos could elicit the recall of autobiographical memory with no significant difference. However, the participants experienced greater pleasure in FIVR than in printed photos. In addition, Yahara et al [60] compared remote and in-person FIVR sessions and found no significant differences in terms of effectiveness.

### Study Limitations

All selected articles but one discussed their study limitations. Common limitations across multiple studies were (1) small sample size [40,52,53,55,57,60], (2) the absence of a control or comparison group [40,52,53,56], and (3) participant selection bias [53,57,58]. Other limitations were related to self-report errors [56,60], the lack of long-term or follow-up assessment [40,52], the unavailability of validated assessment tools for specific components [40], being affected by the COVID-19 pandemic [57,60], exclusion of participants with severe cognitive impairments [57,59], and imperfect FIVR contents [53,55].

### Future Research Directions

The authors advocated for a well-designed study with control or comparison groups, an adequate sample size, and follow-up or long-term assessment for future research on FIVR reminiscence interventions [25,40,55,56,59,60].

They also identified an extensive range of future research directions, encompassing 16 directions across 6 aspects:

1. Caregiver aspect
  - Investigating training for family caregivers to safely use FIVR for reminiscence sessions [52]
  - Examining the effects of the caregiver in charge [25]
2. Health and behavioral improvement aspect
  - Further investigation of the effects of FIVR reminiscence on psychological or behavioral symptoms, cognitive functions, and quality of life [40,53]
3. Social or engagement aspect
  - Examining the social aspect of FIVR and its application in older adult care settings, at home, or with family members [52,54]
  - Examining how to improve participants’ engagement and adherence to the FIVR intervention [40]
  - Exploring how to develop remote options for FIVR reminiscence interventions to promote use among the individual, caregiver, and family members [60]
4. Operation, implementation, or ethical aspect
  - Establishing a metric to assure successful operation of the FIVR intervention [25]
  - Investigating the barriers for the FIVR implementation and related solutions [40]
  - Identifying ethical ramifications of applying the FIVR intervention to individuals with severe dementia [40]
5. Technical aspect
  - Exploring the possibility of using log data, eye-tracking data, machine learning, and artificial intelligence to assist in the design of future FIVR reminiscence intervention prototype [25,55]
  - Understanding the added value of FIVR in reminiscence interventions [40]
  - Comparing FIVR with traditional forms of reminiscence interventions [53]
  - Investigating the method to combine FIVR reminiscence interventions with the digital mapping of media from public sources or family archives [55]
6. Human–FIVR environment interaction aspect
  - Observing participants’ limb movements during the session to analyze their movements in FIVR [53]
  - Investigate how people living with dementia interact with the FIVR environment [59]
  - Exploring how the FIVR environment can create charming experiences with specific therapeutic effects [59]

## Discussion

### Principal Findings

In total, 11 articles [25,40,52-60] detailing empirical studies on FIVR reminiscence interventions in older adults were selected for this review. All but 1 study focused on individuals with cognitive impairments or dementia. The results showed that the intervention enhanced engagement and reduced fatigue. Although some studies reported positive effects of FIVR reminiscence sessions on participants’ anxiety, apathy, depression, cognitive functions, and caregiver burden reduction, the results were not consistent across all studies. Moreover, FIVR showed overall usability and acceptability with manageable side effects among older adults.

### Effectiveness of FIVR-Assisted Reminiscence Interventions in Older Adults

Overall, the review results demonstrated that FIVR-assisted reminiscence interventions, as a nonpharmacological option, were effective in improving some health- and behavior-related outcomes such as engagement and fatigue in older adults. Their effects on depression, anxiety, and apathy were not consistent. There were no significant impacts on other measures such as cognitive functions or dementia status, quality of life, and loneliness. However, it may be too soon to conclude that FIVR may not help induce further health benefits through reminiscence activities, as only 11 studies with small sample sizes and limited time frames were included in this review. Additional studies are required to provide a clearer understanding of this topic.

### Usability and Acceptability

Most participants in the selected studies felt that the FIVR technology was acceptable and enjoyable. However, a study reported that reminiscence interventions may induce negative memories leading to undesirable feelings or undesirable health results [58], which raised the alarm about the selection of visual content. Otherwise, the side effects of FIVR were deemed manageable, with only a few cases reporting motion sickness. According to Chang et al [62], VR motion sickness includes symptoms such as eye fatigue, disorientation, and nausea, which are often caused by viewing dynamic content. The authors contended that motion sickness usually occurred when a person is immersed in moving VR scenes that yields an illusory perception of self-motion but their body is still in a static (standing or sitting) state. Other causes of motion sickness may be related to the fidelity of the VR stimuli and human factors [62]. Although human factors such as genetics and brain reactions may not be easy to change, the fidelity of VR stimuli can be improved, for example, by increasing the resolution or realistic sense of the visual content. There are many web-based platforms offering rich VR resources such as YouTube and Google Street Views, which were adopted by some selected studies. However, this type of content may not be ideal considering some quality problems such as distortion, glare, and low resolution. Several studies selected for this review used this content as stimuli but did not discuss quality issues in their articles.

### Impact on Caregivers’ Burden

Globally, there is a serious shortage in the nursing workforce; consequently, people living with dementia or mild cognitive impairments or other chronic diseases are usually cared for by informal caregivers, most of whom are family members. The care time was approximately 27.1 hours per week per person in 2021 [39], which is a burden on caregivers, especially those who provide informal care but still need to work for a living. This burden may be exacerbated as the person experiences further decline in cognitive functions along with an increase in behavioral problems and the loss of independent living. The selected studies revealed that caregivers viewed FIVR-assisted reminiscence as beneficial and valuable for people living with mild cognitive impairments and dementia. The effects of such interventions on anxiety and apathy may potentially lower caregivers’ burden and companion time. Additional functions that link other people with the people living with dementia remotely through web-based platforms can better involve more family members or friends in reminiscence, which also helps reduce caregiving burden. However, as the hardware and the activity need to be set up and a facilitator is often required during the process, the reminiscence intervention may instead become an additional burden to caregivers, especially those serving in institutional care facilities where a nursing staff member provides care to multiple people.

### FIVR Versus Other Technologies

In the previous section, we outlined the timeline for the different technical aids used in reminiscence interventions. Since the mid-2010s, VR has gradually become a viable option for facilitating the recall of past experiences in older adults. This evolution began with nonimmersive VR [21,27,63], displaying the virtual world through an electronic screen such as a tablet, television, or computer screen. Semi-immersive VR emerged later as another option [23,24], offering a more realistic experience by using multiple large screens or a VR Cave Automatic Virtual Environment that forms an enclosed or semienclosed environment in which to display VR content. FIVR has been considered more effective in evoking memories and is therefore more favorable for reminiscence compared with other aids, because of its fully immersive features. However, this assumption was not fully supported by evidence from the selected studies. It was noted that the results were mixed when comparing FIVR with other technical aids. For example, Saredakis et al [57] and Xu and Wang [59] found that participants preferred using FIVR for visual content over a flat screen or printed photos, whereas participants in another study reported that FIVR did not provide richer reminiscence experiences than those from an iPad [47]. In addition, FIVR may present some challenges for the participant or caregiver in controlling and manipulating the technology [58]. Nevertheless, FIVR is a relatively new technology and its application in reminiscence interventions is still in its infancy. Therefore, it may be too soon to conclude that FIVR is superior or inferior to the other traditional aids. The suitability of using FIVR may depend on the type of reminiscence intervention, content of the visual stimuli, and specific person in the session.

### FIVR for People With Mild Cognitive Impairments and People Living With Dementia

Most of the selected studies focused on individuals with mild cognitive impairments or people living with dementia, making their results applicable to these groups. Only 1 study compared the FIVR effects between the population with mild cognitive impairment and that living with dementia [52]. The results indicated that people living with dementia were more immersed in VR, possibly because the realistic environment captured their attention better than it did for those with mild cognitive impairment. This is important because a higher level of immersion may lead to more emotional engagement, which would trigger unpleasant memories or emotions, resulting in adverse outcomes. Moreover, individuals with mild cognitive impairments displayed greater kinetic engagement during FIVR sessions, potentially owing to better physical health and mobility. However, the exact reasons remain unclear. Notably, there’s a scarcity of studies comparing patients with mild cognitive impairments or dementia with noncognitively impaired groups. Further research comparing multiple user groups, such as young adults, healthy older adults, patients with mild cognitive impairments, and people living with dementia, is essential.

### Types of FIVR Reminiscence Interventions

Only 1 study specified its intervention type as semistructured simple reminiscence. The other 2 types of reminiscence, life review and life story review, were not examined in the selected studies. Our speculations are as follows: (1) life review and life story review require professionally trained staff, posing challenges for research teams; (2) these approaches might demand more time (≥45 min), and using VR goggles for over 20 minutes could induce negative side effects; and (3) most research teams were led by design or computer science professionals, focusing on developing and testing the FIVR prototype. Life review and life story review are vital for end-of-life care, particularly for patients with cancer [15]. Consequently, further research on FIVR-supported life review is critical to address this gap.

### Limitations

The limitations of this literature review are related to the characteristics of the studies and the nature of the scoping review. As noted earlier, the selected studies had some common limitations. First, the sample sizes were relatively small, ranging from 2 to 43 participants (mean 17). Only 4 studies had 20 or more participants. A small sample size has been a common issue in studies involving older adults with cognitive impairment, possibly because of the difficulties in recruitment and retention. Second, most studies were quasi-experiments, case studies, or qualitative research. Only 2 studies used control groups. Third, most studies evaluated the short-term but not the long-term effects of the interventions. Fourth, because most of the studies were conducted by the teams that created the FIVR tools, the study foci were largely on usability aspects but not on health outcomes, although these were also measured.

We attempted to identify related articles through different databases; however, the number of included studies was small (N=11). Possible reasons were that FIVR was a relatively new technology that took some time to be developed and evaluated in this field and that some studies may have been published in non–English language journals. Furthermore, there may have been publication bias; studies that did not find any significant results may not have been published. In addition, the selected studies used various research designs, sample sizes, and measurements, which presented challenges for a systematic, rigorous review, or meta-analysis.

### Implications

#### Additional Future Research Directions

In addition to the research directions raised in the selected articles, we identified the following topics, based on this scoping review results, to further address current research gaps: (1) evaluating the impacts of FIVR quality in terms of fidelity and resolution on the effectiveness of reminiscence sessions; (2) identifying the most effective types of FIVR content, including events, locations, objects, people, sounds, and music; (3) developing and testing feasible strategies to lower caregiver burden through FIVR reminiscence interventions; (4) evaluating a specific type of FIVR reminiscence (simple reminiscence, life review, or life review therapy); and (5) comparing the effects of FIVR reminiscence on multiple user groups, such as young adults, healthy older adults, people with mild cognitive impairments, and people living with dementia. Attention to these research topics would advance our knowledge in this field.

#### Recommendations for Practice

##### Overview

FIVR has become more affordable and accessible over the last decade and therefore could be used in any reminiscence activities led by therapists, certified professionals, caregivers, and family members. It is a relatively safe therapeutic option if developed and implemented appropriately. On the basis of the review results and what we learned from our ongoing pilot studies, we provide a list of recommendations for those who would like to create FIVR stimuli to be used in reminiscence activities:

##### FIVR Content

Content can come from a variety of resources including web-based video sharing platforms such as YouTube and Vimeo, self-produced VR media including photos and videos, and Google Street View images. VR content can be location or event based. These locations can be childhood homes, hometowns, churches, and neighborhoods, whereas events can be weddings, birthday parties, and anniversary ceremonies. The literature suggests that selecting the most familiar places or events or those with pleasurable memories may help maximize positive outcomes. The most recent models of 360° camera allow people to produce high-quality (4K+ resolution) VR photos and videos on their own. Common camera brands include Instra360, GoPro, and Ricoh, with prices ranging from US $200 to US $800 in 2022. To reduce the possibility of motion sickness, we recommend using a fixed camera, which means that the camera is not moving around while taking videos. Once 360° photos and videos are taken, they can be uploaded to YouTube or Vimeo with minimal editing effort. Another type of VR stimulus, computer graphic–based animations or images, is not recommended for public use because it requires well-trained professionals and considerable time to build the content.

##### FIVR Device

We recommend consumer-grade VR goggles for reminiscence interventions because they require minimal technical support and have a less steep learning curve compared with professional goggles. As shown in the selected studies, Oculus was the go-to brand for FIVR reminiscence. The newest model in 2022 is the Oculus Quest 2, which offers its own operating system and can be used independently without connecting to computers or game consoles. It provides easy access to different websites and apps, including YouTube, and VR videos stored in video memory cards.

##### Types of Reminiscence Intervention

Among the 3 types of reminiscence interventions introduced in this paper, simple reminiscence is the least structured and the easiest to carry out, whereas life review and life review therapy may require specific training for the facilitator. Depending on the goals of the intervention and the facilitator’s ability, a decision about the type of intervention to be used should be made collectively by the person and the person’s caregiver, family members, and health care providers for maximal benefit.

##### Facilitators

We support the claim by Molinari [9] that reminiscence interventions can be conducted by both laypersons and trained professionals. In addition to providing cues during the activity, the facilitator should be able to set up the device, assist the person with accessing the VR scene, provide surveillance to avoid possible side effects including motion sickness and negative emotions, and evaluate the effectiveness of the intervention.

It should be noted that users should double-check existing practices or patient safety guidelines before applying these recommendations.

### Conclusions

The growing number of older adults living with dementia or mild cognitive impairments has amplified the crises of caregiver shortage and continuously rising health care costs. We reviewed existing empirical studies that evaluated FIVR reminiscence interventions. The results indicated that such interventions, if delivered appropriately, may help improve the user’s psychological and mental health and potentially decrease the burden on caregivers. The studies also revealed a positive experience with FIVR technology, which most participants found acceptable and enjoyable. On the basis of the literature review, we identified directions for future research and provided recommendations for practice in FIVR reminiscence interventions.

## Appendix

Multimedia Appendix 1 PRISMA-ScR (Preferred Reporting Items for Systematic Reviews and Meta-Analyses extension for Scoping Reviews) checklist.

Multimedia Appendix 2 Article search and screening process.

Multimedia Appendix 3 Original data and data descriptive summary.

## Abbreviations

EUROHIS-QOL: European Health Interview Survey–Quality of Life
FIVR: fully immersive virtual reality
PRISMA: Preferred Reporting Items for Systematic Reviews and Meta-Analyses
PRISMA-ScR: Preferred Reporting Items for Systematic Reviews and Meta-Analysis extension for Scoping Reviews
VR: virtual reality

## Data Availability

The original data are available in Multimedia Appendix 3 [25,40,52-60].
